# Are Physicians Ready for Patients With Internet-Based Health Information?

**DOI:** 10.2196/jmir.8.3.e22

**Published:** 2006-09-29

**Authors:** Farah Ahmad, Pamela L Hudak, Kim Bercovitz, Elisa Hollenberg, Wendy Levinson

**Affiliations:** ^4^Department of MedicineSt. Michael’s HospitalTorontoONCanada; ^3^Department of Public Health SciencesUniversity of TorontoTorontoONCanada; ^2^St. Michael’s Hospital and Department of MedicineUniversity of TorontoTorontoONCanada; ^1^Centre for Research on Inner City HealthSt. Michael’s HospitalTorontoONCanada

**Keywords:** Internet, Gealth information, Consultation, Physician-patient communication, Qualitative, Focus group

## Abstract

**Background:**

An increasing number of patients bring Internet-based health information to medical consultations. However, little is known about how physicians experience, manage, and view these patients.

**Objective:**

This study aimed to advance the understanding of the effects of incorporating Internet-based health information into routine medical consultations from physicians’ perspectives, using a qualitative approach.

**Methods:**

Six focus groups were conducted with 48 family physicians practising in Toronto. The data were analyzed using qualitative methods of content analysis and constant comparison, derived from grounded theory approach.

**Results:**

Three overarching themes were identified: (1) perceived reactions of patients, (2) physician burden, and (3) physician interpretation and contextualization of information. Physicians in our study generally perceived Internet-based health information as problematic when introduced by patients during medical consultations. They believed that Internet information often generated patient misinformation, leading to confusion, distress, or an inclination towards detrimental self-diagnosis and/or self-treatment. Physicians felt these influences added a new interpretive role to their clinical responsibilities. Although most of the physicians felt obliged to carry out this new responsibility, the additional role was often unwelcome. Despite identifying various reactions of patients to Internet-based health information, physicians in our study were unprepared to handle these patients.

**Conclusion:**

Effective initiatives at the level of the health care system are needed. The potential of Internet-based health information to lead to better physician-patient communication and patient outcomes could be facilitated by promoting physician acknowledgment of increasing use of the Internet among patients and by developing patient management guidelines and incentives for physicians.

## Introduction

Internet access is rapidly changing the landscape of health information. In North America, 80% of the general population currently accesseshealth information on the Internet for themselves, family, or friends [1,2]. Moreover, the number of patients bringing Internet-based health information to physicians is on the rise [3,4]. Patients report that use of Internet-based health information enhances their understanding and their ability to manage their health conditions [2,5,6]. Patients also report increased confidence in their interactions with physicians when they are equipped with Internet information [5]. The revolution in health information is having a profound impact on how patients and physicians interact. How are physicians experiencing and managing this shift? What is their view of patients who bring Internet-based health information to the office?

Surveys of physicians have explored the impact of Internet-based health information on physician-patient relationships [7,8]. In a US study involving a nationally representative sample of 1050 physicians, Murray et al reported that 38% believed that the use of Internet information by patients has a beneficial effect on the physician-patient relationship, while 54% reported no effect [7]. A minority of physicians (8%) reported a worsening of the relationship due to physicians feeling “challenged” by patients. Likewise, an online survey by Potts et al with 800 Web-literate physicians concluded that benefits to patients from Internet use outweigh the harm, but that it presents more problems than benefits for doctors [8]. These studies demonstrate that some physicians experience difficulties with “Internet-informed” patients, but it is not clear whyphysicians feel challenged or report more problems [7]. Furthermore, we know little about howphysicians view patients who take the initiative to introduce Internet-based health information into medical consultations. Thus, our goal was to use a qualitative approach to advance the understanding of the effects of incorporating Internet-based health information into routine medical consultations from physicians’ perspectives.

## Methods

### Focus Groups

A series of focus groups with family physicians was designed to explore physicians’ opinions of and experiences with patients who brought Internet-based health information to routine medical consultations. Focus groups allow for participant interaction, and, hence, they create a cueing phenomenon which leads to greater insight as to why certain beliefs and opinions are held. This unique feature of focus groups is not found in face-to-face interviews or questionnaires [9].

The focus groups were co-facilitated by a trained moderator and a member of the research team using standard moderation techniques [10] and an open-ended discussion guide that concentrated on the effect of Internet-based health information on physicians’ interactions with patients. Physicians were also encouraged to discuss the Internet-based health information as (1) enhancing effective use of consultation time, (2) an aid to collaboration between patients and physicians and (3) a challenge to medical authority. All focus groups lasted approximately 2 hours.

### Recruitment

Participants wererecruited by telephone by a local recruitment firm whose database contains demographic information on more than 50000 persons in the greater Toronto area. This firm maintained a registry of health professionals volunteering for research. The study was approved by the Research Ethics Boards of St. Michael’s Hospital and York University. All participants provided written informed consent and were compensated with a modest sum for their time, in keeping with local standards for focus groups.

### Analysis

The focus groups were tape-recorded and transcribed verbatim. The data were analyzed using qualitative methods of content analysis and constant comparison, derived from grounded theory approach [11]. This method aims to identify relevant themes and categories to summarize and systematize the content of the data. This method can effectively capture the range, diversity, and relative importance of certain ideas over others. The analytic process began inductively and was iterative, starting with the identification of key categories for individual questions. Categories were revised as new data and relationships emerged. Finally, categories were organized to reflect overarching messages or themes that spanned individual questions and focus groups [12]. Members of the research team met regularly to discuss the evolving categories and to establish consensus.

## Results

Six focus groups were conducted with 48 family physicians, with an average of 10 participants per group (range 8-12), between April and October 2002. Participants, of whom 54% were male, had been practising for 6 to 27 years, and were encountering approximately 125-149 patients per week on average. All physicians had active practices in the greater Toronto area.

Three overarching and interrelated themes were identified: (1) perceived reactions of patients, (2) physician burden, and (3) physician interpretation and contextualization of information. Within the theme of interpretation and contextualization, the sub-themes of physician roles, resistance, and strategies were identified (Table 1).

**Table 1:** Physician perspectives on Internet-based health information introduced by patients

**Theme**	**Description**
Perceived reactions of patients	Physicians discussed various reactions of patients to the Internet health information brought to medical consultations. Some patients were perceived to have emotional reactions (confusion or distress) concerning the information they read. Others were perceived to have used the information for self-education or for self-diagnosis, with or without self-treatment; the latter group was perceived as challenging.
Physician burden	The introduction of Internet health information into the medical consultations was generally perceived as a burden, attributed to uncertainty about the website validity, limited Internet skills and/or access to up-to-date resources, lack of incentives, and time constraints.
Physician contextualization and interpretation	i) New role Physicians perceived that a new interpretive role was added to their clinical responsibilities when patients introduced the Internet health information into the medical consultations. Although most of the physicians felt obliged to carry out this new responsibility, the additional role was often unwelcome based on the reasons described above. This was further compounded by perceived difficulties in interacting with challenging patients who made erroneous self-diagnoses and/or treatment plans based on Internet health information. ii) Resistance The new role was viewed as a particular burden for older physicians, compared to recent graduates. iii) Strategies Physicians discussed various strategies to cope with the new role. These approaches reflected a collaborative (eg, recommending reliable websites, asking for a follow-up visit) and defensive (eg, referring patients to specialists, suggesting extra charge for time) stance towards the new role.

Dominant views as well as provocative dissenting views are presented below for each theme. Support for our interpretation is provided by including particular quotations from the data that most clearly illustrated the analytic points. From here onward, we refer to Internet-based health information as “Internet health information.” The abbreviations “FG 1,” “FG 2,” “FG 3,” and “FG 4” refer to the four focus groups, and “pg” indicates the page location of the quotes in the transcribed files.

### Perceived Reactions of Patients

Physicians distinguished various patient reactions to the Internet health information brought to medical consultations. Broadly, some patients were perceived to have emotional reactions (confusion or distress) to the Internet health information, while others were perceived to have used the information for self-educationon pre-established medical conditions or for self-diagnoses with or without self-treatment. The latter group was discussed as challenging despite its small size.

Patients with emotional reactions were perceived as being either “confused” or “distressed.” Physicians attributed patient confusion to their limited ability to evaluate, personalize, and interpret abundant Internet health information. Physicians identified these patients as needing clarification of the information brought to the visit:

FG 3, pg 2 Patients who do come with information, I find they are more confused than anything else and they come for clarification.

FG 2, pg 2 They [patients] are getting full of rather stupid facts in many cases, which they do not know how to interpret, which are usually misinformation.

In other instances, Internet health information resulted in patient distress, which was perceived by physicians as patient “anxiety,” “worry,” “nervousness,” panic,” or the patients feeling ‘overwhelmed” or “sicker.” For this cluster of patients, physicians attributed their distress to such factors as the sheer volume of Internet health information, blind faith in or acceptance of Internet data (ie, believing everything one reads), and/or the inability to critically evaluate the personal relevance of the information:

FG 4, pg 3 They are bringing up sort of obscure articles and stuff about different conditions, and some of them are pretty scary.… They think everything is happening.

FG 6, pg 11 It makes them sicker, because they get too worried about what their problems are.

Physicians favorably perceived those patients who used Internet health information for educating themselves about their pre-established medical conditions. The self-educators were perceived to introduce the Internet information into the medical visits for confirmation, without challenging physicians’ expertise.

FG 5, pg 4 I think there’s one situation where the Internet is useful. If the person has the diagnosis, and they want to find out more, educate themselves…, I find that’s actually helpful in cases where…it’s not time-consuming for me.

Patients were perceived as “challenging” when they used Internet information for self-diagnosis or self-treatment or to test the knowledge of physicians. The Internet was deemed simply another potential source of misunderstood health information for the challenging patients who were also described as adversarial, professional, difficult, or neurotic. Some physicians perceived these patients as lacking trust in their provider. Physicians often discussed having to defend their diagnosis or treatment plans, with feelings ranging from anger to frustration (for further details, see Physician Contextualization and Interpretation, below). However, a few physicians discussed how patients felt distressed and needed help after making self-misdiagnoses.

FG5, pg 10 If they’re, however, using it to diagnose, then I think that’s where the problem lies….

FG 4, pg 14 You may disagree with whatever disease that they’ve come in with all this research on.... I think it’s like putting the cart before the horse.... They’re ahead of you and not on the right track.

FG 5, pg 9 I find that they are testing me on how up-to-date I manage to be.

FG 1, pg 5 Part of the therapeutic relationship is the trust and the belief that the doctor will make you better. You don’t have that, you have lost a great portion of your therapy.

### Physician Burden

Physicians discussed several difficulties arising from the introduction of Internet health information into the medical consultations. Expressions such as “awkward,” “tough spot,” “hard time,” “headache,” “nightmare,” “annoying,” “irritating,” and ‘frustrating” indicate the magnitude and nature of the difficulties and the accompanying sense of burden such information placed on physicians.

Concerns about the quality and quantity of health information on the Internet were common. Physicians linked their uncertainty about websites to their lack of information about recommended health sites andthe instability of websites over time.

FG 1, pg 2 I can’t answer for a lot of their questions about the validity of the sites that they’ve received information from….

FG 1, pg 3 I would like to be able to send them to a site that I know is, has reliable information. And I’m not at a point where I have that yellow page book for sites that are approved or somehow controlled.

Time constraint was a major issue for these physicians. They discussed having limited time to deal with Internet-derived “volumes of pages” or “scrolls” of questions that patients bring to their visits. In only a few instances did physicians think Internet health information could actually be time-saving. “Big lists” of questions were particularly problematic and a cause for cynicism among some physicians:

FG 5, pg 13 As soon as that list comes out I panic…[because of] time constraints and everything else.

FG 1, pg 3 I do not mind patients coming in with information, but it is very hard if they present you with a package of, you know, 60 sheets.... Time is really at a premium, so it makes it very difficult.

Furthermore, some physicians acknowledged their limited Internet skills and attributed this to a lack of time to advance their computer skills. This was predominantly discussed among older physicians who seemed reluctant to spend time on learning this new technology:

FG 5, pg 8 One of the frustrations is, knowledge takes time, and it’s fairly busy, in a busy practice, to just keep up and current in all areas.

FG 3, pg 10 All the graduates [are] now using these technologies. So, it’s not that it’s too expensive for us as physicians. It’s that we are caught in this transition in terms of “I do not feel comfortable, the time to learn it.”

There were, however, a few instances of “rare conditions” and “travel medicine” when physicians thought that Internet information brought by patients into medical consultations had been helpful in making a diagnosis or identifying an appropriate referral.

FG 6, pg 12 We [family physicians] do not know everything. Then, it can’t be challenges, actually “teach me” sometimes.

FG 1, pg 3 I had a patient…I didn’t know the diagnosis, something getting off a ship and having vertigo and some problems that ensued. The ENT doctor did not know…and in her search on the Web she found the diagnosis and found a single physician in Ontario. She ended up getting a referral by me….

### Physician Contextualization and Interpretation

Many physicians viewed putting Internet health information in context for patients (ie, providing perspective on information in relation to a patient’s unique history and health status) as part of their responsibility and role. Physicians generally believed they were in the best position to explain, synthesize, and contextualize information because of their training:

FG 6, pg 7 I think for many patients they don’t have the wherewithal to assimilate this sort of information and come up with the appropriate response.... Part of our role is to explain that to them.... They don’t have the background knowledge that we have in order to put it into proper perspective.

The specific roles of physicians in relation to the contextualization and interpretation of Internet health information varied depending on the responses of patients to that information. For those patients perceived by physicians as self-educators, the work of the physicianwas generally limited and sometimes actually reduced.

However, for distressed or confusedpatients who took an uncritical stance toward the information, physicians discussed having the significant task of educating them by putting the information into its proper context. Physicians perceived this task as time-consuming, and, hence, a burden on their routine clinical responsibilities:

FG 5, pg 1 Similar experience where the patients are coming informed with information from the Internet, and sometimes from good sources and sometimes from more anecdotal, personal Web pages where the information may not be entirely correct. Then, you have to do lots of damage control and try to not disinform but try to undo and re-educate.

For patients who used Internet for self-misdiagnosis or self-treatment*,*physicians described doing substantial work in justifying and, at times, even defending their own diagnosis and treatment recommendations or in “debunking” incorrect information. In having their expertise challenged, some physicians felt they were at risk of “losing face” and/or being “put on the spot”:

FG 4, pg 3 Some of my patients come in with a diagnosis...convinced in their minds that this is what they have. Then you’re almost put on the defensive sometimes as to why you think otherwise, or why maybe they should be looking elsewhere for what are their symptoms. So, it’s more of a challenge.

### Resistance

Importantly, not all physicians embraced the role of interpreter, and there were indications of resistance from some to discussing Internet health information with any patient:

FG 3, pg 1 Most of them [patients] know it’s annoying to me when they do it [bring in Internet downloads], so they don’t.

FG 3, pg 3 I just sort of stick with what I know and what I do and how I practice.

The excerpt above highlights the fact that, in some instances, not only is the physician resistant to Internet health information, but his or her patients are aware of this resistance. Older physicians seemed more resistant:

FG 3, pg 11 A lot of people do not take new patients. So, we are going to grow old with our patients. And they’re gonna get used to our ways and we’re gonna [get] used to theirs.

FG 5, pg 14 I think a lot of it is dependent on the age of the physician…. The older physicians are paternalistic and…do not feel comfortable when a patient comes in with an article….

### Physician Strategies

Physicians discussed various strategies they had adopted in order to cope with Internet health information introduced by patients during medical consultations. These strategies included recommending reliable websites, asking for a follow-up visit, or expressing limited knowledge on specific details:

There is nothing wrong with saying, “You know what? I do not see a lot of this but I am going to find out for you.” (FG 1, pg 15)

They have huge time commitment and an emotional commitment to whatever it is they’ve brought in. So, I’ll say, “Leave it with me for a couple weeks and let me think about it.” And I’ll usually look it over, probably not while they’re there, not that minute. (FG 4, pg 5)

Notably, some physicians discussed strategies of “firing” the patient, referring patients to specialists, or charging for extra time. These strategies have the potential to undermine the physician-patient relationship.

FG 4, pg 9 Well, frankly, we’re paid for [the] visit. So, if your patient [is] having a $15 visit, you’re not going to sit for 15 minutes going through all this, you’re going to get them out of the office.

FG 3, pg 4 If they come in and it’s too much and it’s too specialized…I let them slug it out with the specialist. They’re paid very special money to do this kind of work.

FG 4, pg 13 Maybe we can bill our patients privately for extra time to review research with them, if we can choose to do that or not do that.

FG 5, pg 7 They’re coming back [with Internet information]. It requires a little looking into. If you’re tired, of course, you’ll probably just fire them…if they’re really belligerent.

## Discussion

Physicians in our study generally perceived Internet health information as problematic when brought by patients to medical consultations. They believed that Internet information generated patient misinformation, leading to confusion, distress, or an inclination towards detrimental self-diagnosis and/or self-treatment. Physicians felt these undesirable but common influences of Internet health information added a new interpretive role to their clinical responsibilities. Although most of the physicians felt obliged to carry out this new responsibility, the additional role was often unwelcome. Despite identifying various reactions of patients to Internet health information, physicians in our study were unprepared to handle these patients.

Despite the patient-perceived benefits of bringing Internet health information into medical consultations [2,5,6], physicians in our study viewed such consultations as too demanding. First, physicians viewed the task of contextualizing and interpreting the informationas time-consuming. Misinformed, confused, and distressed patients needed not only an empathetic ear, but also supplementary education on how to assess the quality and relevance of Internet health information. In addition, intense involvement was deemed necessary for the challenging patients who used Internet information for self-diagnosis and/or treatment. Hence, physicians felt powerless when faced with the task of fulfilling their clinical responsibilities as well as answering a series of questions concerning Internet health information. Second, physicians experienced emotional difficulty in interacting with the challenging patients who made erroneous self-diagnoses and/or treatment plans based on Internet health information. Some physicians interpreted these situations as a threat to their medical expertise. The physicians’ perception of threat also seemed to have a ripple effect to other patients who just needed clarification of the Internet health information but who encountered physician reservations.

Perceptions of consultations being too demanding were further compounded by physicians’ uncertainty about website validity, alack of incentives to contextualize the Internet health information for patients, and limited access to up-to-date resources. The scepticism expressed by physicians about the quality of health information on the Internet is in accordance with existing empirical studies [13]. Nevertheless, the rising use of the Internet among patients to obtain health information [14] calls for concrete measures to facilitate physicians’ access to up-to-date technology and listings of reliable websites. In our study, the lack of tangible incentives appears to have been a fundamental barrier for physicians taking up the role of contextualizing and interpreting Internet health information. This led physicians to cope by making referrals to specialists—an expensive solution if increasingly adopted. Likewise, some physicians declined to continue caring for,or charged extra money to, patients who brought Internet health information to medical visits. These less than optimal strategies could undermine the continuity of the physician-patient relationship, which is a concern as continuity with the same health care provider is highly valued by patients [15] and primary care practitioners [16].

In our study, physicians’ perceptions of difficulties in adopting their new role of contextualization and interpretation seemed to vary according to their perception of patients’ reactions to Internet health information. This possible inter-relatedness should be examined in future research.

### Implications

Many academic and nonacademic institutions have recently begun to train health care providers to critically evaluate Internet materials available to patients [17]. However, the un-preparedness of the physicians in our study to undertake the contextualization and interpretation of such information indicates the limited effectiveness of current efforts. In light of the study findings, we propose several possible avenues of improvement.

First, there is a need to increase the awareness of health care providers about the Internet-generated “reversed” information asymmetry [18]. Today, patients have easy access to medical information, and expert knowledge is no longer a “prohibited” zone for the general public. Such awareness would alleviate physician apprehension and the perceived threat to their medical expertise upon seeing a patient with Internet health information. Formal and informal educational initiatives for health care providers need to foster acknowledgment and, hence, acceptance of the emerging norm as increasing numbers of patients bring Internet health information to medical visits. Information management is a recognized task of physicians [19]. Internet health information is changing the dynamics through which this task is activated.

Second, training programs for health care providers need to enhance physicians’ understanding of patients’ perspectives on Internet information. For instance, patients with serious sickness are more likely to ask their physician about Internet health information [20]. Also, patients who feel overwhelmed by Internet information report difficulties in making an informed decision about their own care [21]. Physicians need to be prepared to address alternative sources that patients learn from, including the Internet. It may be useful for medical experts and health service administrators to establish patient management guidelines for physicians seeing patients with Internet health information. Such an approach has been applied to address issues around email communication between health professionals and patients [22]. The guidelines for management of patients with Internet health information should be sensitive to the diverse needs of patients. The guidelines should include avenues for physicians to have ready access to up-to-date lists of recommended health websites, or the ‘yellow page” resource, described by our study participants. In addition, guidelines may incorporate a team approach to meet patient needs. For instance, nurse practitioners and diet counsellors routinely educate patients with respect to lifestyle modifications and self-management of chronic conditions. These existing human resources could be mobilized to address the patient misinformation, confusion, and distress generated by Internet health information.

Third, time-pressed physicians require tangible incentives to undertake the new role of contextualization and interpretation. Alongside monetary incentives, which require progressive structural changes in health care services, other incentives targeting professional “pride” should be considered. These include recognition in the form of certificates, award nominations, or credits for continuing medical education on information technology. The incentives should particularly target those health care providers who graduated beforethe inclusion of information technology in health care training programs.

Finally, patient-focused strategies related to Internet health information could complement physician-patient communication. Health care institutions could actively develop general patient guidelines on how to optimize the usefulness of Internet health information in physician-patient encounters. However, educating the public to apply evaluation criteria in a critical appraisal of the health information available on the World Wide Web, as proposed by others [23,24], is an overly optimistic approach. This approach ignores the existing digital divide among various strata of the population in accessing and understanding the Internet health information [25]. A cautious approach to health promotion via the Internet is recommended to avoid reproduction of existing social divisions [26-28]. Hence, too much emphasis on promoting the “responsible*”*use of the Internet among patients entails an inherent risk of ignoring less resourceful people.

### Limitations

The study has some limitations. We used a convenience sample of urban physicians, which limits the generalizability. Physicians in our study seemed to have highly negative attitudes toward the influence of Internet health information on physician-patient relationships compared to prior physician surveys. Possible explanations include the cuing phenomenon of focus group methodology, use of prompts in the discussion guide, metropolitan sample of family physicians, and/or individual characteristics of the participants, such as number of years since graduation. Future research studies should examine physicians’ perceptions by speciality, geographic location, and practice years. Nevertheless, our study findings represent the tip of the iceberg.

### Conclusion

The dramatic increase in patient access to Internet health information of varying quality influences physician-patient relationships. We identified several factors that will need to be addressed in order for this information to be optimally integrated. Effective initiatives at the level of the health care system are needed. The potential of Internet health information to lead to better physician-patient communication and patient outcomes could be facilitated by promoting physician acknowledgement of an increasing use of Internet health information by patients and by developing patient management guidelines and incentivesfor physicians.
